# Molecular Dynamics Simulation of Thin Silicon Carbide Films Formation by the Electrolytic Method

**DOI:** 10.3390/ma16083115

**Published:** 2023-04-15

**Authors:** Alexander Galashev, Ksenia Abramova

**Affiliations:** 1 Institute of High-Temperature Electrochemistry, Ural Branch of Russia Academy of Science, Academicheskaya Str., 20, Yekaterinburg 620990, Russia; 2 Institute of Chemical Engineering, Ural Federal University Named after the First President of Russia B.N. Yeltsin, Mira Str., 19, Yekaterinburg 620002, Russia

**Keywords:** silicon carbide, electrodeposition, thin film, molecular dynamic, molten salt, structure

## Abstract

Silicon carbide is successfully implemented in semiconductor technology; it is also used in systems operating under aggressive environmental conditions, including high temperatures and radiation exposure. In the present work, molecular dynamics modeling of the electrolytic deposition of silicon carbide films on copper, nickel, and graphite substrates in a fluoride melt is carried out. Various mechanisms of SiC film growth on graphite and metal substrates were observed. Two types of potentials (Tersoff and Morse) are used to describe the interaction between the film and the graphite substrate. In the case of the Morse potential, a 1.5 times higher adhesion energy of the SiC film to graphite and a higher crystallinity of the film was observed than is the case of the Tersoff potential. The growth rate of clusters on metal substrates has been determined. The detailed structure of the films was studied by the method of statistical geometry based on the construction of Voronoi polyhedra. The film growth based on the use of the Morse potential is compared with a heteroepitaxial electrodeposition model. The results of this work are important for the development of a technology for obtaining thin films of silicon carbide with stable chemical properties, high thermal conductivity, low thermal expansion coefficient, and good wear resistance.

## 1. Introduction

Silicon carbide (SiC) has long attracted attention as a material with a wide range of unique properties, such as high mechanical strength, thermal, and radiation resistance, due to strong Si-C bonds in the material [1]. SiC is a semiconductor material with a wide bandgap. It exhibits chemical inertness as well as being characterized by a strong breakdown field, high thermal conductivity, and high-saturated electron velocity. In nature, silicon carbide exists in several crystalline modifications—polytypes—such structures are characterized by different stacking sequences of Si-C layers [2]. Each modification is characterized by a set of properties that makes it the most preferable application in a particular area. For example, amorphous a-SiC:H films have broad application prospects in the design of solar cells as a p-element, the inclusion of material in the design of batteries makes it possible to influence the optoelectronic properties of the product [3,4]. The 3C-SiC polytype has the smallest band gap among all others and it is also the most thermally stable modification [5]. Polytype 3C-SiC—a modification with a cubic crystal lattice—is used in transistor technology. Hexagonal modifications of silicon carbide: 4H-SiC, 6H-SiC are widely used for growing other polytypes on their surfaces, or as substrate materials in the design of solar cells, lithium-ion batteries, microelectronics objects, etc. [6].

Obtaining high-purity silicon carbide today is an important technological challenge. At the same time, Thin film technologies are used in various fields of industry and the main requirements that apply to them are low cost, simplicity of the equipment used, short deposition time, and flexibility in terms of the shape on which the film is produced. The majority of thin-film methods require complicated equipment [7]. The method of physical deposition from the vapor phase is characterized by a rather high deposition rate. However, it is difficult to control the thickness of the deposited films here. Moreover, contamination is possible in the film by the container material. The method requires sophisticated equipment, and is required to be carried out in a vacuum or inert gas. High-quality thin films are created by laser ablation. However, this is a very expensive method with a very slow settling rate. Plasma spraying is characterized by high productivity and the possibility of obtaining a variety of coatings. This is a very expensive method, usually with a low settling rate. The sputtering method includes thermal evaporation and pulsed laser deposition. Electrically conductive materials, such as metals, are being sprayed by DC sputtering; radio frequency (RF) sputtering is performed for nonconductive targets. A common method is magnetron sputtering, in which positive ions present in a magnetically enhanced glow discharge plasma bombard a target. The sputtering process is quite slow and expensive compared to vacuum spraying.

The method of chemical vapor deposition (CVD) is widely used. Within the framework of CVD technology, the decomposition of precursors on the substrate surface can be carried out by various methods, for example, using plasma (PECVD). One of the advantages of CVD methods is the possibility of applying films of uniform composition and thickness. In this case, the substrate may have a complex configuration. In addition, this method is distinguished by the simplicity of process control and the possibility of quick adjustment when changing the substances used, and the high chemical purity of the precipitated product. However, the cost of CVD equipment is high and film growth is very slow.

The method of electrodeposition from molten salt represented by this model has a number of advantages over other methods for producing thin films [8]. Among the advantages of the method, one can note a high film deposition rate (about 1 μm per min), and the possibility of obtaining multilayer films with control over the thickness of each layer, which depends on time and change in the applied voltage. In addition, the method does not require expensive equipment and is flexible in choosing the substrate material and its shape.

Films are increasingly being used to replace conventional bulk materials. In this way, better performance, greater flexibility, lower volume and weight, and lower cost are achieved [9]. Films based on SiC applications in diodes, thin film transistors (TFT), and micro-electro-mechanical systems (MEMS) devices [10,11].

The application of a SiC coating by a relatively low-temperature electrolytic deposition method is of considerable interest in the production of metal-silicon carbide composite materials. Such materials have increased strength, high thermal conductivity, and low coefficient of thermal expansion. Metals potentially applicable as a matrix for graphene and SiC inclusions include Ag, Cu [12,13], and Ni [14]. In the case of electrolytic deposition, it is possible to avoid the problem of alloy formation of metals with carbon and achieve a uniform distribution of SiC nanoparticles over the substrate surface [15].

However, regardless of the method of obtaining film, the question of the material’s defective structure remains important. For example, when growing Si films on the surface of 4H-SiC, numerous defects on the substrate surface lead to a nonuniform thickness and, as a result, to a nonuniform distribution of stresses in the resulting silicon film [16]. The study of the initial stages of the formation of SiC films can be useful in describing the defective structure of the material. Computer modeling is a powerful tool for studying the electronic, mechanical, physical, and chemical properties of materials, as well as for describing the processes occurring in materials both at the macro- and nano-levels.

A wide range of industries in which silicon carbide films are used makes it important to develop models that describe the process of obtaining SiC films and the degree of influence of the substrate material on the structure of such films. It is also important to refine existing models.

The aim of this work is to develop a molecular dynamics computer model that allows for simulation of the initial stage of SiC film growth during its electrolytic production both on metal and non-metal surfaces. In addition, the effect of various substrates on the structure and difference in the mechanisms of the SiC film growth must be also determined, and the role of the potential interaction during the SiC growth on a graphite substrate must be established.

## 2. Materials and Methods

At the initial stage, the geometrical model of the molecular dynamic (MD) system was created: KF, KCl, and KI crystals were placed above the substrate surface, and the substrate was fixed (i.e., the substrate atoms did not participate in thermal motion). Copper, nickel, and graphite were considered substrates. The dimensions of the system were 7 × 7 × 11 nm, and the total number of atoms varied depending on the chosen substrate: 13,794 for copper and nickel substrates, and 19,312 for a graphite substrate. The composition of the salt melt was constant for all systems: 1728 atoms in the KF crystal (19.2 mol %), 864 in the KCl crystal (9.6 mol %), and 6400 atoms in the KI crystal (71.2 mol %). The percentage of components in the electrolyte was chosen in accordance with the experimental data on the electrolytic production of silicon [17]. The nickel and copper substrates included five layers and faced the melt with the (001) plane, the graphite substrate was hexagonal α-graphite and included four layers of graphene. In the horizontal (0*x*, 0*y*) directions, periodic boundary conditions (PBC) were applied; fixed boundary conditions (FBC) acted in the vertical direction (along 0*z*).

At the next stage, the potential interaction acted in the MD system was described. The action of Lennard–Jones potential with the Coulomb term in the melt is determined by the expression:

(1) $$\Phi \left(r_{ij}\right)=\frac{Z_{i}Z_{j}}{r_{ij}}+4\varepsilon \left[{\left(\frac{\sigma }{r_{ij}}\right)}^{12}-{\left(\frac{\sigma }{r_{ij}}\right)}^{6}\right],$$

where *Z_i_* is the charge of the *i*-atom, *r_ij_* = |*r_i_* − *r_j_*| is the distance between atoms *i* and *j*, *ε* is the depth of the potential well, and *σ* is the distance at which the interaction between the *i* and *j* atoms becomes zero. Charges of the system components are: *Z_K_* = +1, *Z_Cl_* = −1, *Z_F_* = −1, Z*_I_* = −1.

The interaction of deposited Si and C particles with the metal surface was described by the Morse potential:

(2) $$\Phi \left(r_{ij}\right)=D_{ij}^{o}\left[\exp\left\{-2\alpha_{ij}\left(r_{ij}-r_{ij}^{o}\right)\right\}-2\exp\left\{-\alpha_{ij}\left(r_{ij}-r_{ij}^{o}\right)\right\}\right],,$$

where *D*_o_ is the depth of the potential well, *α* is the parameter that determines the “rigidity” of the bond, and *r*_o_ is the equilibrium bond length.

The interaction parameters for pair potentials are presented in Table 1 and Table 2.

The particles of Si and C had an electric charge of +4e and interacted with the ions of the salt melt in accordance with the Coulomb law. Within the framework of the classical molecular dynamics approach, we considered two possible variants of the interaction arising between the deposited particles and the graphite substrate. In the first case, an interaction was described as a pair long-range using the Morse potential; in the second case, an interatomic interaction was described as a many-particle short-range interaction using the Tersoff potential [19].

It is known that a covalent bond is formed between Si and C particles, this bond depends on the local environment and solid angles; therefore, the use of the many-particle Tersoff potential seems natural when describing the interaction between a silicon carbide film and a graphite substrate. However, during the electrolytic coatings production, the presence of various defects in the obtained films is inevitable, and the concentration of defects can reach ≈ 10^13^ cm^−2^ [20]. Thereby, when modeling such polytopic structures as SiC, it is important to take into account their defectiveness and choose the correct potential to describe the interaction in the film. The many-particle Tersoff potential is short-range, i.e., the cutoff radius of potential is from 3 to 4 Å, and the lattice constant of silicon carbide compounds varies within 3.1–4.38 Å [21] depending on the polytype. Thus, the choice of a short-range potential may not be justified when describing the defect structure of silicon carbide films. The Morse potential was cut off at a much larger distance (10 Å).

**Figure 1:**
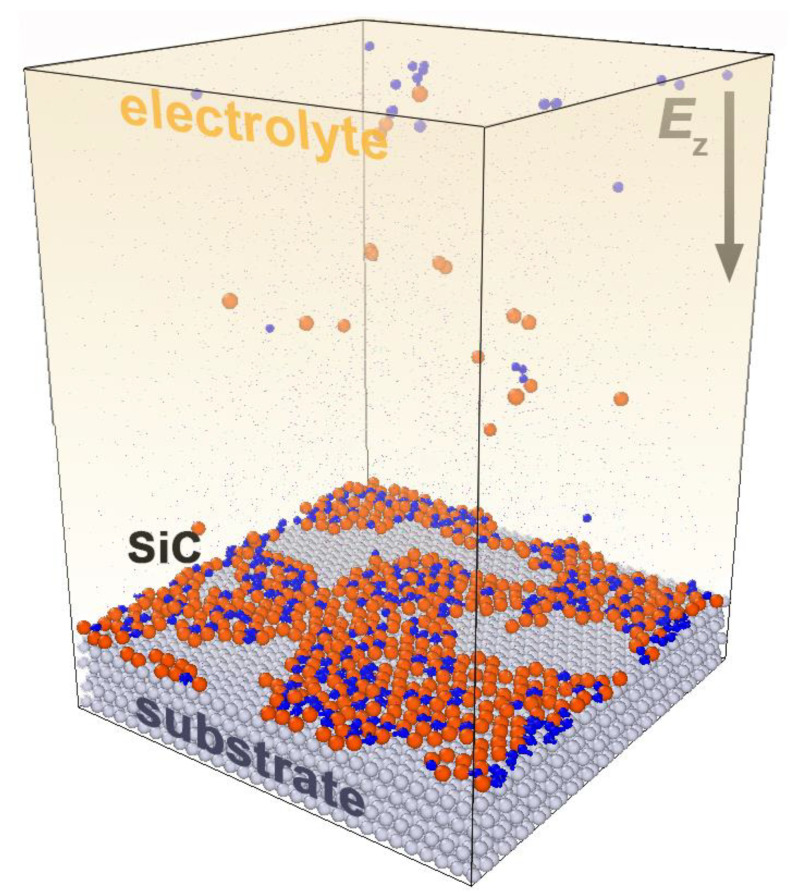
Process of Si^4+^ and C^4+^ ions deposition from the melt onto the substrate; the ions were introduced periodically, and moved under the action of an external field *E*_z_.

The deposition was carried out by alternately introducing Si and C particles into the system, which moved to the substrate under an electric field *E*_z_ = −10^4^ V/m, where the minus indicates the field direction relative to the outer normal (Figure 1). A new particle appeared in the system every 30 ps, i.e., 30,000 ∆*t*, where ∆*t* = 1 fs. The deposition process was considered to be completed when most of the substrate surface was covered with a SiC film. The temperature of the KF-KCl-KI melt was 1000 K, which coincides with the experimental conditions for this class of systems [22].

The detailed structure of the obtained films was studied by the statistical geometry method based on the construction of Voronoi polyhedra (VP) [23,24]. The metric characteristic in this method is the angular distribution of the nearest geometric neighbors. To construct the angular distributions, the angles *θ* formed by all pairs of neighbors with a vertex in the center of VP are calculated. The number of VP faces determines the number of neighbors of the central atom. The topological characteristics are the distributions of VP over the number of faces and the distribution of VP faces over the number of sides. Structural analysis based on the construction of VP provides more complete three-dimensional information about the structure than a one-dimensional radial distribution function. In addition, due to the difference in the heights of the deposited atoms, it is possible to use three-dimensional rather than two-dimensional VPs for structural analysis. The distribution of VP over the number of faces establishes the probability of finding a given number of nearest geometric neighbors around the central atoms. The distribution of VP faces over the number of sides indicates the probability of finding *m*-membered structural rings when viewed from the center of the VP in the directions of the location of the nearest geometric neighbors.

All these statistical distributions were obtained at the final stage of growth (at *t* > 20 ns) of the SiC film. VPs were plotted for each particle (both Si and C) on the substrate. The construction of VPs was carried out every 300 ps. So, for conditional 500 particles, 33,300 polyhedra were built in 20 ns.

Techniques, such as CVD and Sputtering, to obtain silicon carbide require the generation of toxic Si-containing vapor, ultra-high purity precursors, templates, or catalysts [25,26]. Using electrosynthesis in molten salt, one can directly convert solid metal oxides into micro/nanostructured metal/alloy powders, spending only electrons on the reduction process [27,28]. The advantages and disadvantages of our method in comparison with other main methods for obtaining thin SiC coatings can be seen in Table 3.

## 3. Results

**Figure 2:**
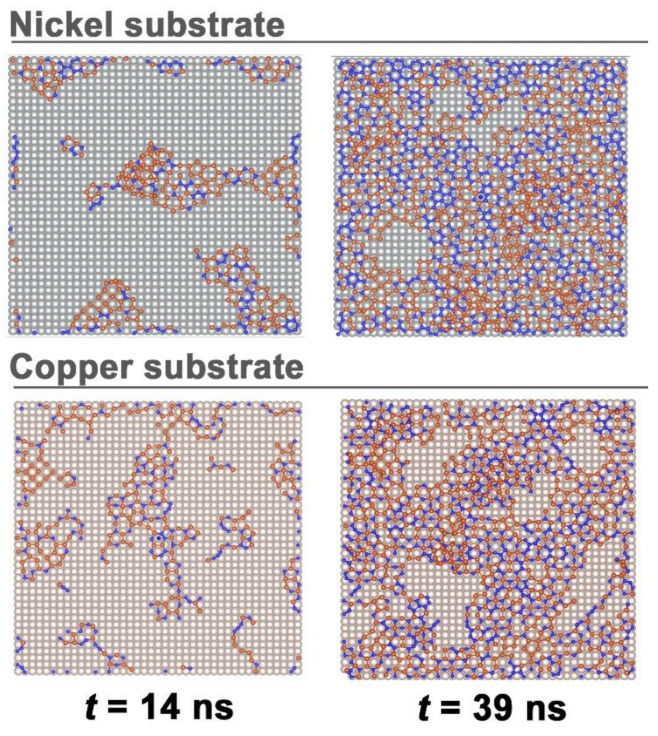
Stages of growth of SiC films on metal substrates: nickel (**top**) and copper (**bottom**); in the figures on the right, the SiC film largely covers the corresponding substrate.

Figure 2 shows the successful formation of SiC films on metal substrates. As can be seen from the figure, SiC islands formed on nickel and copper substrates have different morphologies. At the early stage of growth (*t* = 14 ns), the growing and already rather large fragments of the SiC film on the Ni(001) substrate have a more compact shape with fairly smooth boundaries, while the growth of the SiC film on the Cu(001) substrate occurs with the formation rather large fragments with jagged boundaries, as well as small fragments of a sinuous shape, including those in the form of curved chains of Si and C atoms. By the time of 39 ns, a defective SiC film was formed on both substrates. Moreover, in the case of the Ni(001) substrate, only three large defects (cavities) remained in the film, while on the Cu(001) substrate there are at least twice as many such defects. Consequently, in the latter case, a greater part of the substrate is freer from Si and C atoms than in the first case. Since the total number of Si and C atoms deposited on both substrates is approximately the same, islands with multilayer deposition of Si and C atoms on a copper substrate either occupy a larger area or such SiC buildups have a greater height than in the case of a nickel substrate. In other words, the growth of subsequent film layers with an incomplete first layer on a copper substrate proceeds more intensely than on a nickel substrate.

**Figure 3:**
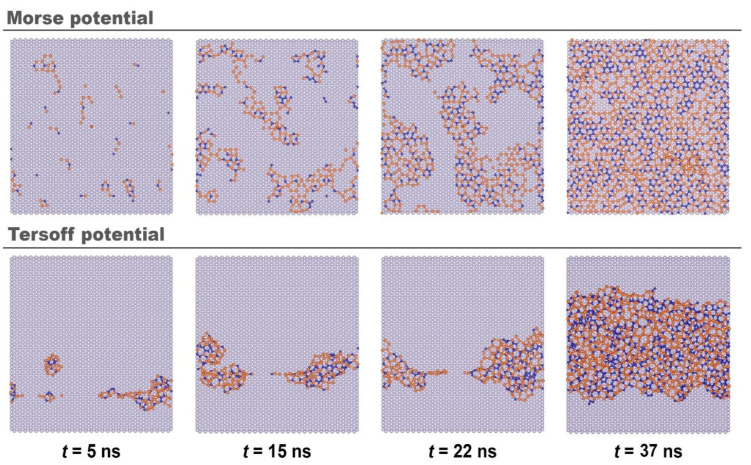
Stages of growth of SiC films on a graphite substrate in the description of the “film-substrate” interaction by the Morse potential (**top**) and Tersoff one (**bottom**).

The final result of the SiC film formation on a graphite substrate largely depend on the nature of the interaction between the deposited atoms and the substrate. Figure 3 shows the stages of growth of a SiC film on graphite for various types of interaction between film and substrate. Already at the initial stage, one can note significant differences in the mechanisms of formation of silicon carbide films, depending both on the type of substrate and on the shape of the potential. In the case of using the Morse potential on a graphite substrate, a picture similar to metal substrates is observed: at the initial time, several stable clusters are formed at the surface, and new particles coming from the melt attach more actively to the substrate, forming new film growth centers. In this case, the growth of the initially formed clusters slows down. Clusters do not remain immobile; by the time of 15 ns, they not only increase in size, but also move relative to their original position. 

A different picture is observed when the Tersoff potential is used to describe the “film-substrate” interaction (Figure 3). As shown in Figure 3, the growth mechanisms of SiC films are different. In the case of the Tersoff potential, a single growth center is formed, which is weakly bound to the substrate surface and has high mobility. Three-dimensional growth in such a center begins before the SiC film covers the entire substrate surface. In the case of the Morse potential, a uniform coating with a small number of defects is formed on the graphite surface.

**Figure 4:**
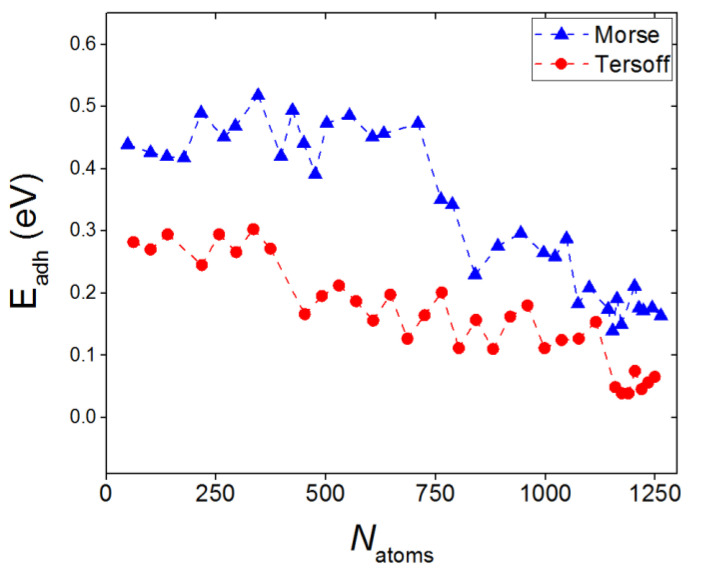
Adhesion energy between SiC films and a graphite substrate as a function of the total number of silicon and carbon atoms at the substrate surface; the values are given for the cases when the “film-substrate” interaction was described by the Morse potential (marked in blue) and the Tersoff potential (marked in red).

A significant difference in the mechanisms of SiC film formation when describing their interaction by two types of potentials manifests itself in the difference of adhesion values *E_ad_* between the film and substrate. Figure 4 shows the dependences *E*_ad_(*N*_atoms_) for systems described by two types of potentials. It can be noted that the use of the Tersoff potential to describe the interaction between the film and the substrate leads to a decrease in the adhesion energy by almost a factor of two. In the case of the Morse potential, it can be seen that the adhesion energy to the substrate decreases abruptly when there are more than 750 Si and C particles in the system. This is due to the fact that individual clusters are contracted into a single SiC film at the surface, which leads to a decrease in the adhesion of the film to the substrate. The force of attraction between dissimilar surfaces, which creates adhesion, is determined by expression

(3) $$E_{\mathrm{adh}}=\frac{E_{G-\mathrm{SiC}}-E_{G}-E_{\mathrm{SiC}}}{N_{\mathrm{atoms}}}$$

where $E_{G-\mathrm{SiC}}$ is the energy of the graphite substrate + SiC film system, $E_{G}$ is the energy of the graphite substrate, $E_{\mathrm{SiC}}$ is the energy of the SiC film, and $\;N_{\mathrm{atoms}}\;$is the number of all atoms in the system.

In the model under consideration, the main contribution to the change in the adhesion energy comes from the last term in the numerator of expression (3). The Tersoff potential leads to an adequate description of the interaction at a low defectiveness of the resulting SiC film and gives an inadequate high value of the adhesion energy in the case of a highly defective film. As the number of atoms deposited onto the graphite substrate increases, the energy $E_{\mathrm{adh}}$ decreases more rapidly when the Tersoff potential is used to determine it. At a value of $N_{\mathrm{SiC}}$ ≈ 1100, the energies of the formed film are equalized when both potentials are used. However, the morphology of the film in both cases changes when new Si and C atoms are added to it. Therefore, a stable trend in the behavior of the adhesion energy does not appear in either case, and the dependences $E_{\mathrm{adh}}\left(N_{\mathrm{atoms}}\right)$ do not yet intersect at the considered values of $N_{\mathrm{SiC}}$.

**Figure 5:**
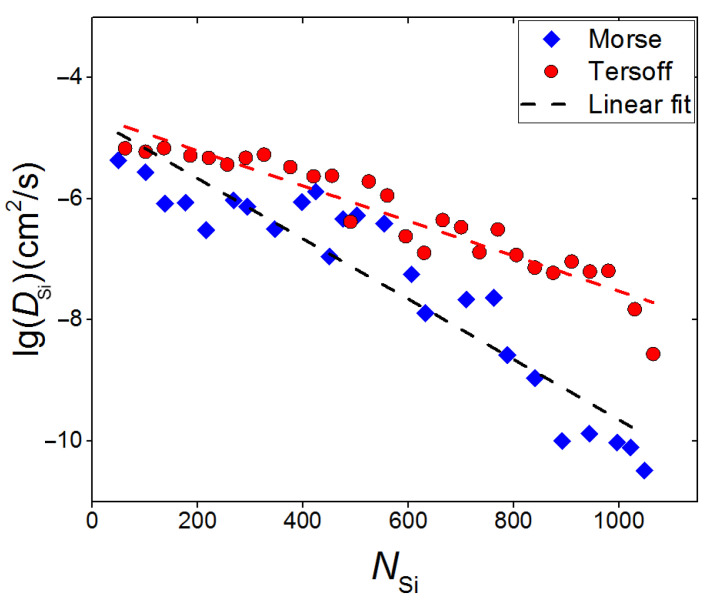
Surface diffusion of Si atoms as a function of the total number of particles located on the graphite substrate; dependences (in a logarithmic scale) are shown for the case of the “film-substrate” interaction by the Morse potential (marked in blue) and the Tersoff potential (marked in red).

The shape of the potential function of particle interaction with the surface also affects the surface diffusion of SiC clusters. Figure 5 shows the dependence of the surface diffusion of Si atoms on the total number of silicon atoms at the substrate surface. In the case of pair potential, it can be noted that at stage 0 < *N*_Si_ < 650, the surface diffusion is about 10% less than in the case of many-body potential (Tersoff); at *N*_Si_ > 650, the difference becomes more pronounced and reaches 30–35% on average. This is quite expected due to the formation of a continuous coating when the Morse potential is used. When a continuous layer has not yet been formed, the value of the diffusion coefficient for a system with a Morse potential is 5.7 × 10^−12^ m^2^/s, in the case of modeling with the Tersoff potential it is 2.4 × 10^−11^ m^2^/s. It is of interest to compare the results of the study of diffusion in this work with the data of our previous work [18], where films of pure silicon were deposited on various substrates, including graphite substrate, using the MD model and Morse potential. The coefficient of diffusion of surface Si atoms during the deposition of pure silicon was *D*_Si_ = 1.46 × 10^−11^ m^2^/s. Thus, in the presence of C atoms among the deposited atoms, the coefficient of diffusion of surface Si atoms decreased by about 2.5 times.

**Figure 6:**
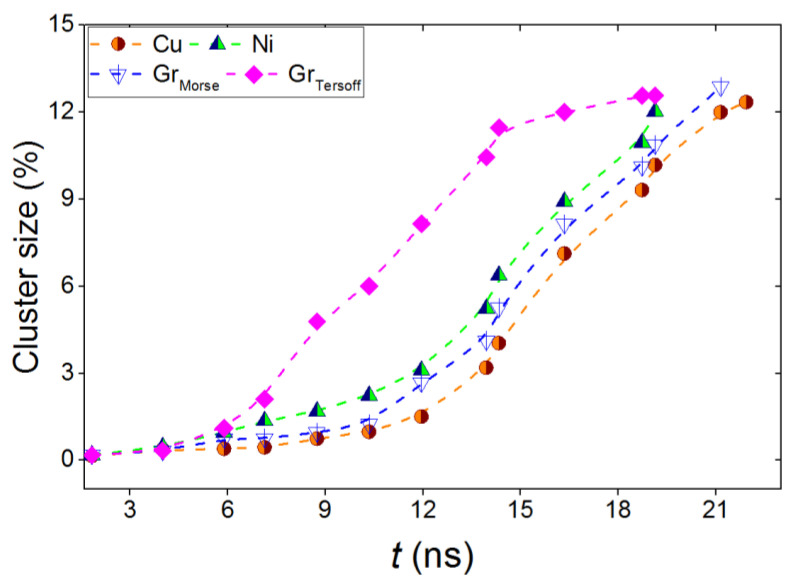
Relative change in the average SiC cluster size for all types of substrates; the blue and pink colors show the dependences for the cases when the SiC-substrate interaction was described by the Morse and Tersoff potentials, respectively.

Figure 6 shows the change in the average size of a SiC cluster with time relative to the total area of the substrate. Dependences were plotted until the SiC clusters contracted on the substrate surface into a continuous coating. It should be noted that with an increase in the average size of the SiC cluster by more than 10% of the total area of the substrate, a continuous SiC coating was formed on its surface. In almost all cases, the film growth proceeds according to the same script: at the initial stage, growth is rather slow; after 12 ns, a sharp increase in the average cluster size is observed; by 18 ns (on nickel) or 21 ns (on copper and graphite), individual islands merge and form a single cover. A different picture is observed on a graphite substrate when the SiC–substrate interaction is described by the Tersoff potential. In this case, intensive growth can be noted from the time of *t* = 6 ns; after 12 ns, the growth slows down, and by the time of 18 ns, a SiC film is formed on the surface. However, unlike other substrates, such a film does not cover the entire area of the graphite substrate; a bulk SiC film is formed on the surface, and the further addition of Si^4+^ and C^4+^ ions occur predominantly to the silicon carbide film, rather than to the substrate.

**Figure 7:**
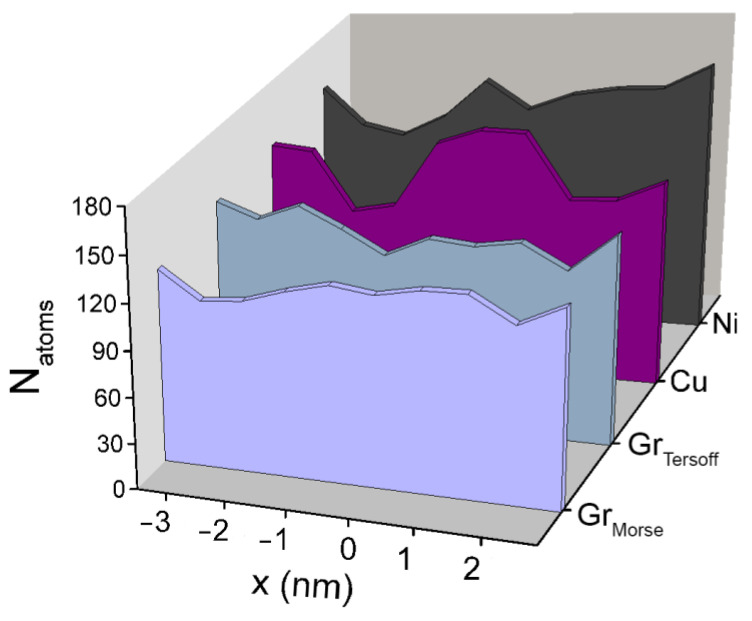
Numerical distribution density of SiC film atoms along the 0x direction for different types of substrates and interactions.

The numerical density of the distribution of atoms in the SiC film along the 0x direction for all types of substrates, and for a graphite substrate with different SiC–substrate potential interactions, is shown in Figure 7. The calculation of these distributions was carried out as follows: the plane of the SiC film was divided into elementary areas and the number of Si and C atoms belonging to each area was counted.

**Figure 8:**
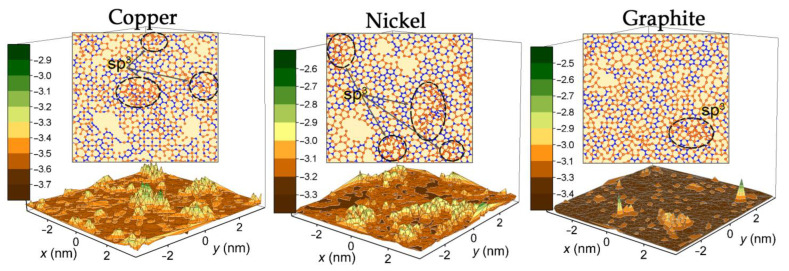
Main view of SiC films obtained on the various substrates, the simulation time for all types of systems is ≈ 40 ns. The color scale on the left represents the relief height of the obtained films. The insets show the *xy*-projections of the corresponding SiC films. Si atoms are marked in orange, and C atoms are marked in blue. Ovals indicate the centers of three-dimensional growth on the SiC film surfaces; the interaction between the deposited atoms (Si, C) and the graphite substrate is described by the Morse potential.

It can be noted that the most uniform distribution of Si and C atoms in the film is observed when SiC is located on a graphite substrate when describing the SiC–substrate interaction by the Morse potential. For metal substrates, the uniformity of the distribution of atoms in films significantly decreases, this is due to the imperfection of such films (Figure 8) and the appearance of three-dimensional growth centers. Such centers were formed at the final stage of modeling for all types of systems. The highest film growth rate and the largest number of three-dimensional growth centers were observed for the nickel substrate. In the case of graphite, when modeling the SiC–substrate interaction with the Morse potential, the area covered by the film turned out to be maximized. The mismatch between the lattice periods of the substrate and the formed SiC film leads to the formation of defects in the films. The smallest lattice mismatch (~7%) refers to the nickel substrate, on which the 3C-SiC cubic modification is formed. The largest discrepancy of SiC and substrate lattice periods corresponds to the graphite substrate. In addition, in this case, the bond between the film and the substrate is the smallest, which leads to the formation of the most defect-free coating.

**Figure 9:**
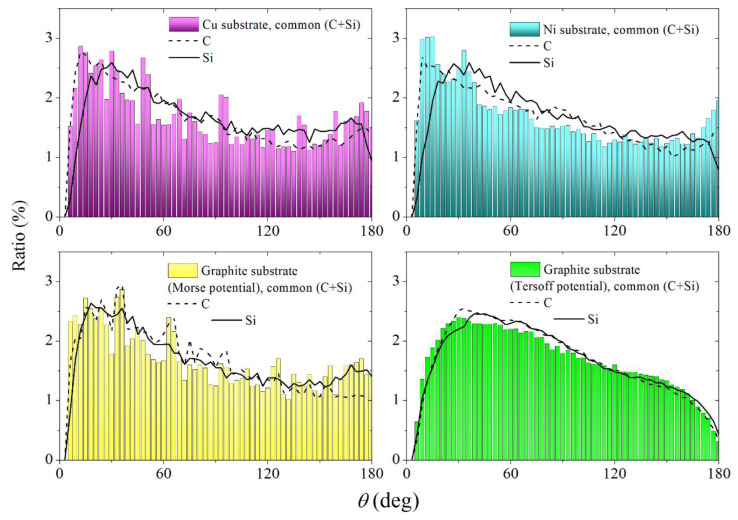
Angular distributions of the nearest geometric neighbors obtained at the final stage of deposition of the SiC film on various substrates, both for the entire SiC film and for its individual components (C and Si); the interaction between Si or C atoms and a graphite substrate is carried out using Morse and Tersoff potentials.

The study of the structure of the obtained SiC film’s short-range order can be performed by the method of statistical geometry based on the construction of Voronoi polyhedra. The polyhedra were constructed for the entire SiC system without taking into account the geometric dimensions of Si and C atoms. The construction of VP was also performed for individual components of this system, i.e., for subsystems of Si and C atoms. Figure 9 shows the angular distributions of the nearest geometric neighbors (θ spectra) for the entire SiC system and for its Si and C subsystems when the film was on copper, nickel, and graphite substrates. In the case of a graphite substrate, θ-distributions were obtained corresponding to different types of interaction between the SiC-film and the substrate. It can be seen that in the case of metal substrates common (Si + C) θ spectra have a higher intensity in the vicinity of angles 0° and 180° than similar spectra obtained on a graphite substrate. The higher intensity of the θ spectra at the edges (i.e., in the vicinity of the 0° and 180° angles) indicates a greater number of triplets of atoms located approximately on the same straight line. This feature is more characteristic of crystalline rather than irregular packings of atoms. The spectra obtained when using the Morse potential to describe the interaction of deposited atoms with the substrate are more jagged than the θ spectrum calculated using the Tersoff potential. The jaggedness of the θ spectrum is most likely associated with the lower surface mobility of deposited atoms and their stronger attraction to the substrate. 

The smooth shape of the θ spectrum obtained with the Tersoff potential, both for the entire (Si + C) system and for its individual parts (Si and C) indicates a high mobility of the atoms on the substrate. Spectra of this type were observed during the thermal destruction of the two-component Lennard-Jones crystal [29]. On the whole, the θ spectra for subsystems turn out to be smoother than for the entire system. This smoothing can be associated with an increase in the distances between atoms during the transition from the entire system to a separate subsystem.

**Figure 10:**
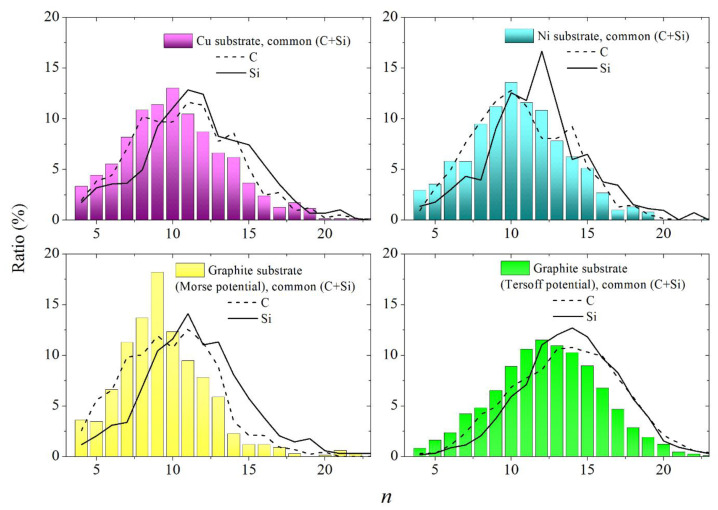
Distributions of Voronoi polyhedra over the number of faces obtained at the final stage of the SiC film deposition on various substrates, both for the entire SiC film and for its individual components (C and Si); the interaction between Si or C atoms and a graphite substrate was carried out using Morse and Tersoff potentials.

Using the Morse potential to describe atomic co-precipitation results in a distribution of polyhedra in terms of the number of faces (*n* distribution) for the entire system, the maximum location of which falls at *n* = 10 for metal substrates and *n* = 9 for graphite substrate (Figure 10). When the Tersoff potential acts during the deposition of atoms, the maximum of the *n* distribution of the complete system shifts towards a larger *n* (*n* = 12). It can be seen from the figure that the maxima of the *n* distributions for the subsystems, as a rule, shifted to the right of the locations of the maxima for the respective systems. The shift to the left of the maximum of the complete system occurs due to the inclusion in the consideration of Si-C bonds, which are not taken into account in the case of subsystems. Significant values of *n* corresponding to the maxima of these distributions indicate the looseness of the quasi-two-dimensional packing of atoms, the difference in the heights of the atom’s location, and the absence of long-range order. In the case of an ideal planar arrangement of atoms, the location of the maximum must be at *n* = 6.

**Figure 11:**
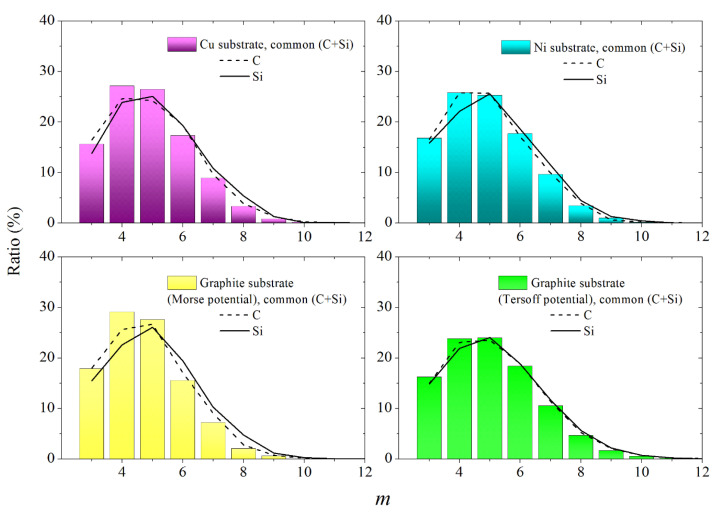
Distributions of VP faces over the number of sides obtained at the final stage of deposition of the SiC film on various substrates, both for the entire SiC film and for its individual components (C and Si); the interaction between Si or C atoms and a graphite substrate was carried out using Morse and Tersoff potentials.

The VP facet distributions over the number of sides (*m* distributions) appear to be very similar for the systems obtained using the Morse potential during the atoms deposition, and only a small significant difference from these distributions can be seen for the *m* distribution calculated using the Tersoff potential (Figure 11). For the first three systems, the maximum of the *m* distribution falls on *m* = 4, while for the fourth system, the location of the maximum is characterized by the value *m* = 5. As a rule, for all subsystems, regardless of the potential used, the maximum of the *m* distribution falls on *m* = 5. Such an arrangement of this extremum indicates the predominance of fifth-order rotational symmetry in the arrangement of atoms on the substrate. The rotational symmetry of the fifth order is characteristic of the irregular packing of atoms [30], while the predominance of the fourth order symmetry is a sign of the crystallinity of the atomic packing. Only in the case of using the Tersoff potential to describe the deposition of atoms on a graphite substrate for both subsystems and the complete system (i.e., in all three cases), the maximum of the *m* distribution falls on *m* = 5. Therefore, this system has the most irregular structure among all considered here systems.

## 4. Discussion

In this paper, we present a model of the electrochemical deposition from molten salt aimed at obtaining thin films of silicon carbide. The method of electrochemical deposition from the molten salt has certain advantages over other methods for obtaining such films, including ion and magnetron sputtering, chemical vapor deposition, pulsed laser deposition, and the sol-gel method [31,32,33,34]. The application of such alternative methods is limited due to the complex equipment and precise experimental conditions, including high vacuum. The advantage of the electrochemical method of growing thin films also lies in the fact that it allows one to control the current, the amount of electricity, and the overvoltage. With this method, one can quantitatively determine the degree of phase transformation, as well as the amount of substance used to form a new phase. By varying the overvoltage, one can control the deposition process to a certain extent [35]. Important process parameters can be established by performing a computer experiment that precedes the actual physical study. In particular, the use of a computer model makes it possible to establish the kinetics of deposition, which must be studied in detail to ensure reproducibility. Previously, computer tests to obtain a silicon carbide film by electrolysis have not been carried out. 

The process of electrochemical phase formation is often represented by simple models [35]. As a rule, they consider two limiting nucleation mechanisms: instantaneous and progressive nucleation. The actual process of film formation turns out to be highly idealized. Instantaneous nucleation assumes that all active centers are filled with deposited atoms almost simultaneously. The growth process occurs due to the attachment of new atoms to stable clusters. The surface of the substrate is usually inhomogeneous. The most active centers of the substrate are first filled with atoms. Accordingly, progressive nucleation suggests that nuclei appear on the surface gradually.

**Figure 12:**
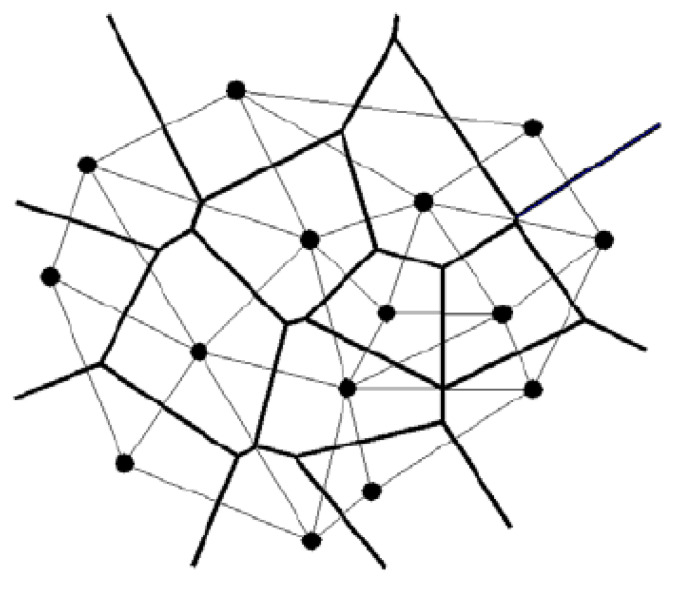
Formation of Voronoi polyhedra by nucleus collision: the bold line network is the Voronoi-diagram reflecting the forming crystals, and the network of thin lines is the Delaunay partition of a two-dimensional system of effective centers of action of the substrate onto the forming film.

In practice, one often deals with the heteroepitaxial electrodeposition, in which nuclei with different azimuthal orientations are formed. As a result of such electrodeposition, a continuous single-crystal layer characteristic of the auto-epitaxy process cannot be obtained. In the case of heteroeitaxy, when the nuclei formed come into contact with the substrate in the same crystallographic plane, they acquire different azimuthal orientations with equal probability. In this case, when a continuous layer appears on the substrate, randomly oriented crystalline regions are formed. In this case, if neighboring nuclei had the same orientation, they merge into a single crystal. The model representation of the device of the obtained polycrystalline film is the partition of the two-dimensional space by the corresponding Voronoi polyhedra, as shown in Figure 12. The edges of the graph shown in the figure by thick lines correspond to the boundaries of the crystals. The vertices of the polyhedra are formed at the meeting points of the edges. Due to the randomness of the distribution of VP nuclei, the vertices turn out to be nondegenerate, i.e., only three edges converge in each of them. Using the Euler relation, which characterizes the Voronoi partition, and the statistical approach to the formation of graph vertices, it is not difficult to obtain the ratio between the number *N*_cryst_ of two-dimensional crystals on the substrate and the number *n*_nucl_ of initial nuclei per unit area, in the form [36]

(4) $$N_{\mathrm{cryst}}\;\approx \;{\left(n_{\mathrm{nucl}}S\right)}^{1/2},$$

where *S* is the area of the electrode.

Circles in Figure 11 show VP centers that can be correlated with the effective centers of influence of the substrate on the formation of two-dimensional crystals. The latter is located on the substrate directly under the VP centers. Note that the effective centers of action of the substrate on the film being formed are not identical to the activation centers on which the nuclei grow. The pairwise connection of effective centers of action by segments perpendicular to the VP edges leads to the appearance of a network formed by triangles, which is illustrated in the figure by thin lines. Such a network on the surface of the substrate is represented as a Delaunay partition of the space occupied by its effective centers of action. The smaller cells in this network result in the formation of a greater number of effective centers concentrated per unit area of the substrate.

Thus, the Voronoi decomposition can be used not only to study the detailed atomic structure of deposited films but also to represent geometrically polycrystalline films formed during the heteroepitaxial electrodeposition. However, the relatively small size of the system and limited computer time did not allow us to study the heteroepitaxial electrodeposition.

Using the method of statistical geometry, we have shown that the degree of crystallinity of the single-layer SiC film is determined not only by the substrate material but also by the nature of the adhesive interaction. Thus, in the case of a deposition of a SiC film on a graphite surface, a higher crystallinity of the film is achieved using a long-range Morse pair potential rather than the manybody but short-range Tersoff potential.

Pang et al. [37] reported on the obtaining of silicon carbide by the electrolytic method, the CaCl_2_ chloride melt was used as an electrolyte, the electrolysis temperature was 1173, after the completion of the electrolysis process, and a SiC deposit was recorded on the SiO_2_/C cathode. X-ray diffraction was used to determine the phase composition of the electrolytic products, and the morphology and elemental analysis of the obtained samples were controlled using scanning electron microscopy and energy-dispersive X-ray spectroscopy. As a result, the authors obtained data corresponding to the structured crystalline modification of 3C-SiC. Ostwald ripening was identified as the most probable mechanism of coating growth [38,39]: a layer supersaturated with silicon carbide is formed near the cathode (substrate) surface and new incoming particles do not stick together, but dissolve in larger SiC grains on the surface.

Pezoldt et al. [40] discussed surface diffusion and self organization of SiC clusters at the Si surface are discussed. Using the method of molecular beam epitaxy, the authors irradiated the silicon surface with a flow of carbon particles, the substrate temperature was 873–1223 K, and the intensity of the C-flow was constant. The surface of irradiated silicon was studied in detail using an atomic force microscope. The authors note that chains of SiC clusters are formed at the edges of flat areas of the silicon surface. There are a large number of dangling bonds at the edges of such steps, and the motion of C atoms in the longitudinal or transverse direction relative to such steps can be considered as particle jumps between local minima (adsorption sites) on the energy surface of the substrate. The paper presents the calculated values of the activation energy for carbon atoms at the surface. For example, when the step crosses directly on the Si substrate, the activation energy *Ea* is 1.7 eV, and when the particle initially moves along the step and then crosses it, *Ea* = 2.2 eV.

Our work shows that when modeling the SiC-graphite substrate interaction by the Tersoff potential (Figure 3), SiC clusters nucleate at the simulation cell boundary (with operating PBC), then the cluster contracts, forming an extended chain of atoms along the surface of the substrate. 

Films obtained on a graphite substrate are distinguished by a higher density uniformity than films deposited on a metal surface. The appearance of three-dimensional islands during the film growth is largely due to the degree of long-range action of the potential describing the interaction between the film and the substrate. The effect of sp^3^ hybridization of the growing film was also observed on a graphite substrate when a long-range Morse potential was used. However, this effect is less pronounced than that in the cases of the Tersoff potential. The many-body potential is more accurate since it reproduces data consistent with the existing hypotheses regarding the mechanisms of formation of SiC films. Among the main advantages of the method presented here are the following: inexpensive oxides are used as raw materials; the sufficiently low temperature of electrolysis (700–900 °C), which allows for reducing energy consumption; product synthesis is produced with a varied and controlled morphology. The electrolytic method for the reduction of oxides is resistant to a strong decrease in the strength of the applied electric field and, in the limiting case, can be carried out even without the use of electric current (synthesis of nanowires from silicon carbide), which further reduces the cost [41]. The disadvantage of this method is the use of a large amount of molten salt as the reaction medium.

## 5. Conclusions

Electrodeposition from the molten salt is a simple one-stage process that makes it possible to obtain a wide class of functional materials, including SiC. In this work, we have developed a computer model that describes the initial stages of SiC film growth on various substrates. The coefficients of surface diffusion of silicon atoms were calculated when the “SiC-graphite substrate” interaction was determined by both pair and multiparticle potentials. The adhesion energy of the obtained films with a graphite substrate has been established, which can be determined with a large error due to the low values of this value and inadequate presentation of the “film-substrate” interaction. For systems where the deposition was performed on metal substrates, the growth rate of SiC clusters was estimated. The numerical density of the obtained films was calculated both on metal and graphite substrates. Molecular dynamic modeling has shown that single-layer SiC films obtained on copper and nickel substrates are characterized by a high level of defectiveness and a low level of density uniformity. At the same time, the deposition of silicon carbide on a graphite substrate, performed at two different potentials describing the interaction between the SiC film and the substrate, and showed a significant dependence of the structure of a single-layer film structure on the type of adhesive interaction. Our molecular-dynamic model can contribute to a deeper understanding of the initial stages of electrochemical phase formation, as well as to the expansion of conceptions about the silicon carbide film formation mechanisms on metal and non-metal surfaces. It can be expected that on this basis, an environmentally friendly, energy efficient, and affordable process for obtaining advanced technical materials will be developed.

## Figures and Tables

**Table 1:** Parameters of the Lennard–Jones potential describing the interaction in the melt. Data from [18].

Ion Pair	*ε* (eV)	*σ* (Å)
K-K	0.0323	4.886
F-F	0.00781	3.12
Cl-Cl	0.00509	4.417
I-I	0.0147	4.009
K-Cl	0.0128	4.6514
K-F	0.0159	4.0029
K-I	0.0218	4.447
F-Cl	0.00631	3.769
F-I	0.0107	3.565
Cl-I	0.00865	4.213
Si-K	0.0253	3.216
Si-F	0.00198	3.9515
Si-I	0.0109	3.876
Si-Cl	0.0124	3.995
C-K	0.00782	3.045
C-F	0.000613	3.781
C-I	0.00596	3.705
C-Cl	0.00385	3.824

**Table 2:** Morse potential parameters describing the interaction of deposited particles with metal and graphite substrates.

Ion Pair *i-j*	*D^o^_ij_* (eV)	*α_ij_* (Å^−1^)	*r^o^_ij_* (Å)
Si-Ni	0.181	4.081	2.032
Si-Cu	0.163	4.049	1.864
C-Ni	1.009	1.987	2.664
C-Cu	0.912	1.957	2.443
Si-C	0.435	4.648	1.947
C-C	2.423	2.555	2.552

**Table 3:** Comparison among some methods used to grow SiC films.

	Electrodeposition from Molten Salts	CVD	PeCVD	Sputtering
Cost	Low	Fair	Fair	Fair
Uniformity	Fair	Fair	Fair	Fair
Substrate versatility	Very good	Good	Very good	Very good
Stress control	Good	Poor	Very poor	Good
Thoughput	Good	Varies	Very good	Fair

## Data Availability

Not applicable.
